# Supplementary material for: B chromosome retrotransposed sequences persist through speciation, contributing to genomic and regulatory innovations in the fish genus Psalidodon (Characiformes, Acestrorhamphidae)
Source: PLoS One. 2026 Jan 2;21(1):e0340085. doi: 10.1371/journal.pone.0340085 (PMC12758807; doi:10.1371/journal.pone.0340085)
Supplement: S1 Fig — F1 = forward primer. R1 = reverse primer 1. R2 = reverse primer 2. (PDF) [file pone.0340085.s001.pdf]

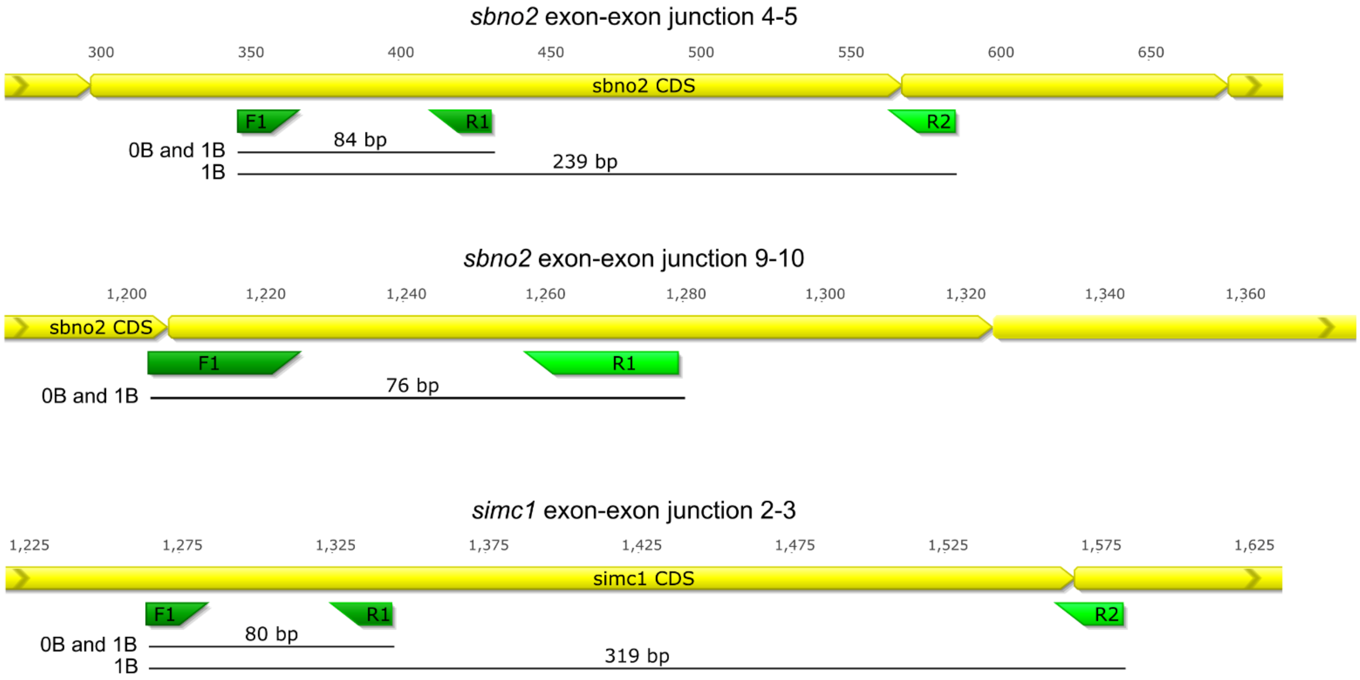

S1 Fig. Illustration of the primer scheme for multiplex and regular PCR to confirm the presence of B chromosome-specific sequences in samples with B chromosomes. F1 = forward primer. R1 = reverse primer 1. R2 = reverse primer 2.
