# Supplementary figures and images for: B chromosome retrotransposed sequences persist through speciation, contributing to genomic and regulatory innovations in the fish genus Psalidodon (Characiformes, Acestrorhamphidae)
Source: PLoS One. 2026 Jan 2;21(1):e0340085. doi: 10.1371/journal.pone.0340085 (PMC12758807; doi:10.1371/journal.pone.0340085)

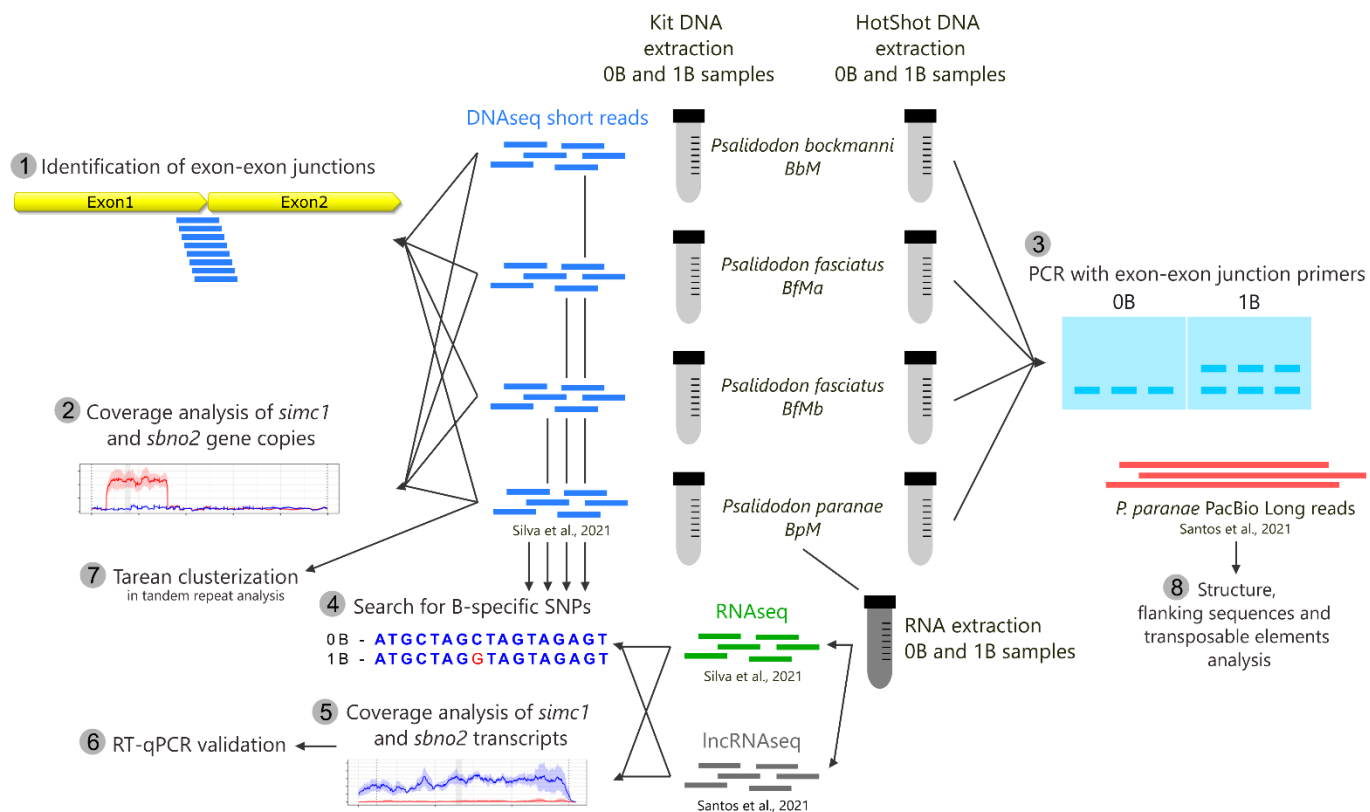

**S2 Fig. Summary of the investigation approach.**

Supplement: S2 Fig — (PDF) [file pone.0340085.s002.pdf]
