# Supplementary material for: B chromosome retrotransposed sequences persist through speciation, contributing to genomic and regulatory innovations in the fish genus Psalidodon (Characiformes, Acestrorhamphidae)
Source: PLoS One. 2026 Jan 2;21(1):e0340085. doi: 10.1371/journal.pone.0340085 (PMC12758807; doi:10.1371/journal.pone.0340085)
Supplement: S3 Fig — (PDF) [file pone.0340085.s003.pdf]

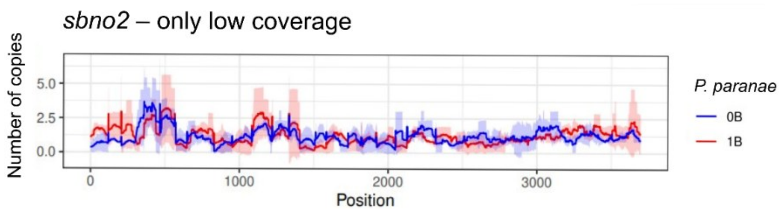

S3 Fig. Coverage graph of the *sbno2* gene on the B chromosomes of *Psalidodon paranae* excluding the high-coverage region to visualize the absence of differences between 0B and 1B samples.
