# Supplementary material for: B chromosome retrotransposed sequences persist through speciation, contributing to genomic and regulatory innovations in the fish genus Psalidodon (Characiformes, Acestrorhamphidae)
Source: PLoS One. 2026 Jan 2;21(1):e0340085. doi: 10.1371/journal.pone.0340085 (PMC12758807; doi:10.1371/journal.pone.0340085)
Supplement: S4 Fig — The high-coverage peaks observed in c, d were removed from the analysis in a, b for better visualization of the low-coverage regions. The shaded areas indicate the exon boundaries. (PDF) [file pone.0340085.s004.pdf]

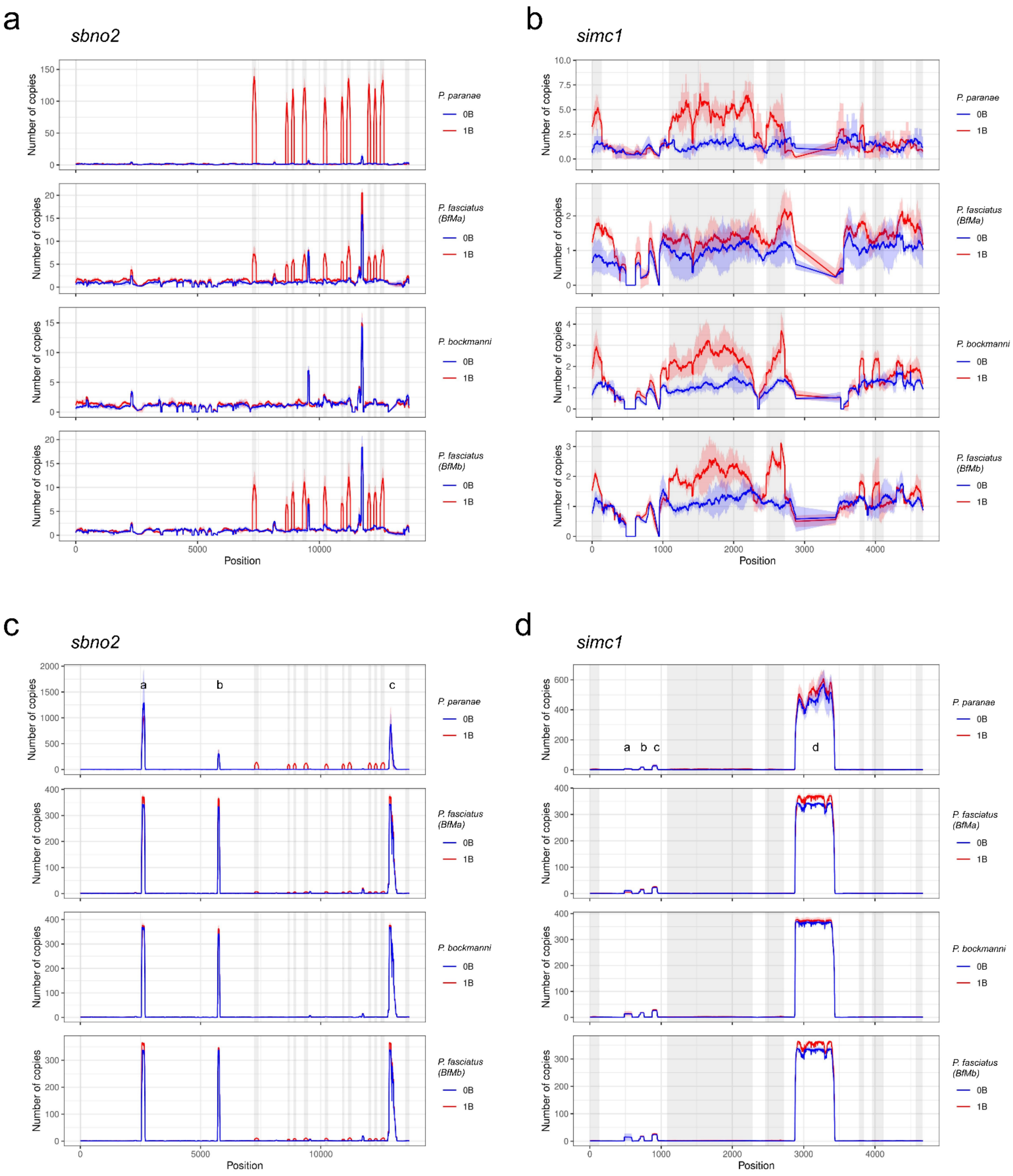

S4 Fig. Coverage graph of the full *sbno2* (a, c) and *simc1* (b, d) gene sequences of *Psalidodon paranae* (including introns). The high-coverage peaks observed in c, d were removed from the analysis in **a**, **b** for better visualization of the low-coverage regions. The shaded areas indicate the exon boundaries.
