# Supplementary material for: B chromosome retrotransposed sequences persist through speciation, contributing to genomic and regulatory innovations in the fish genus Psalidodon (Characiformes, Acestrorhamphidae)
Source: PLoS One. 2026 Jan 2;21(1):e0340085. doi: 10.1371/journal.pone.0340085 (PMC12758807; doi:10.1371/journal.pone.0340085)
Supplement: S5 Fig — (PDF) [file pone.0340085.s005.pdf]

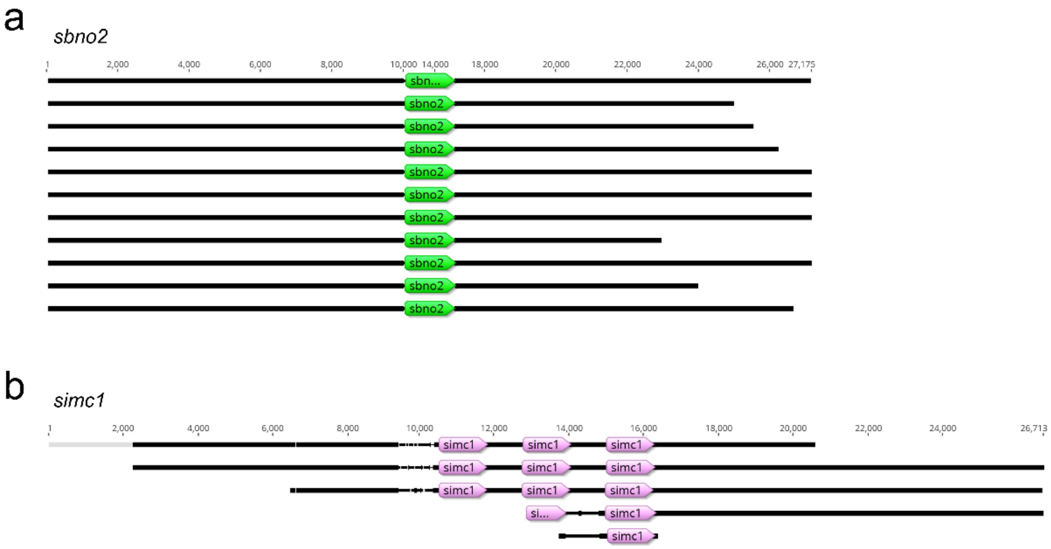

S5 Fig. Example of *Psalidodon paranae* PacBio reads alignment with the pseudogene sequences annotated, *sbno2* (green) (a) and *simc1* (pink) (b).
