# Supplementary material for: B chromosome retrotransposed sequences persist through speciation, contributing to genomic and regulatory innovations in the fish genus Psalidodon (Characiformes, Acestrorhamphidae)
Source: PLoS One. 2026 Jan 2;21(1):e0340085. doi: 10.1371/journal.pone.0340085 (PMC12758807; doi:10.1371/journal.pone.0340085)
Supplement: S6 Fig — The dotted lines show the exon-exon junctions. Note the presence of reads crossing the exon-exon junctions in all the mappings. In b, the red boxes identify the reads crossing the exon-exon junctions for visualization purposes. (PDF) [file pone.0340085.s006.pdf]

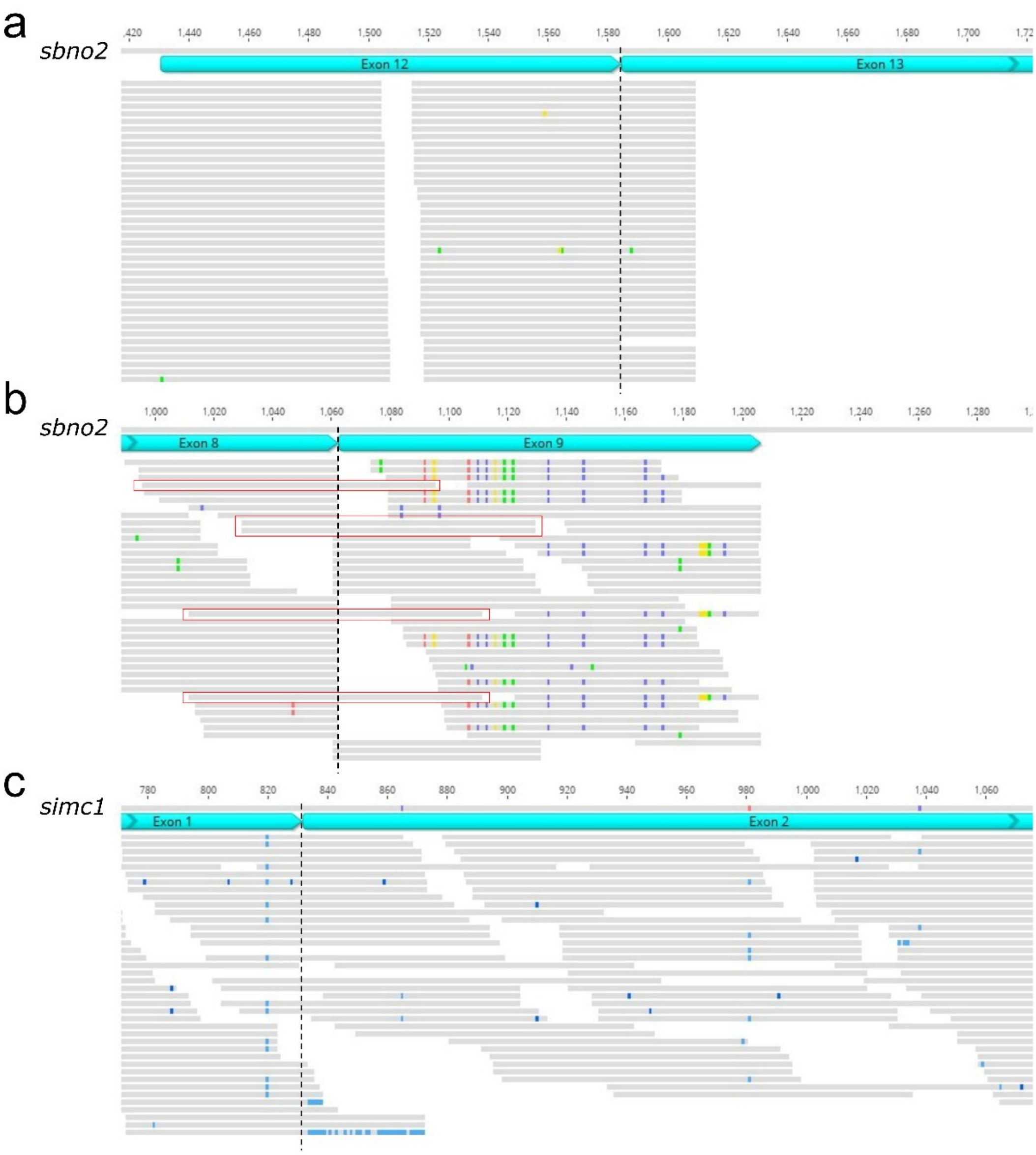

S6 Fig. Example of Illumina short reads mapped to a reference sequence. The dotted lines show the exon-exon junctions. Note the presence of reads crossing the exon-exon junctions in all the mappings. In b, the red boxes identify the reads crossing the exon-exon junctions for visualization purposes.
