# Supplementary material for: B chromosome retrotransposed sequences persist through speciation, contributing to genomic and regulatory innovations in the fish genus Psalidodon (Characiformes, Acestrorhamphidae)
Source: PLoS One. 2026 Jan 2;21(1):e0340085. doi: 10.1371/journal.pone.0340085 (PMC12758807; doi:10.1371/journal.pone.0340085)
Supplement: S7 Fig — The dotted lines show the exon-exon junctions. The sample highlighted in blue showed reads mapped in the exon-exon junctions. Note the slightly higher coverage in the blue sample on exons present in the 0B genome (green boxes) compared to its flanks (exons 7 and 11) and the red sample. (PDF) [file pone.0340085.s007.pdf]

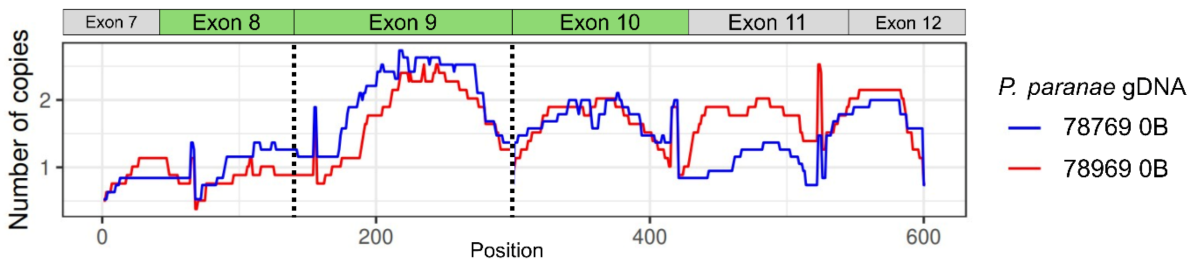

S7 Fig. Coverage graph of *sbno2* using 0B reads of two *P. paranae* individuals. The dotted lines show the exon-exon junctions. The sample highlighted in blue showed reads mapped in the exon-exon junctions. Note the slightly higher coverage in the blue sample on exons present in the 0B genome (green boxes) compared to its flanks (exons 7 and 11) and the red sample.
