# Supplementary material for: B chromosome retrotransposed sequences persist through speciation, contributing to genomic and regulatory innovations in the fish genus Psalidodon (Characiformes, Acestrorhamphidae)
Source: PLoS One. 2026 Jan 2;21(1):e0340085. doi: 10.1371/journal.pone.0340085 (PMC12758807; doi:10.1371/journal.pone.0340085)
Supplement: S1 Raw images — (PDF) [file pone.0340085.s008.pdf]

All images were obtained using a CCD-based imaging system.

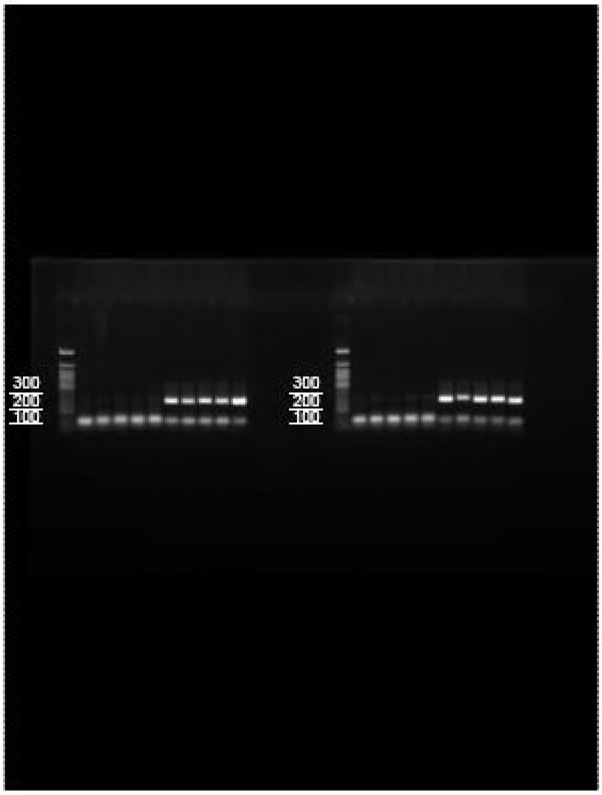

The image above shows, on the left, *Psalidodon paranae* BpM-sbno2, and on the right, *Psalidodon fasciatus* BfMa-sbno2, corresponding to Figure 1b of the article.

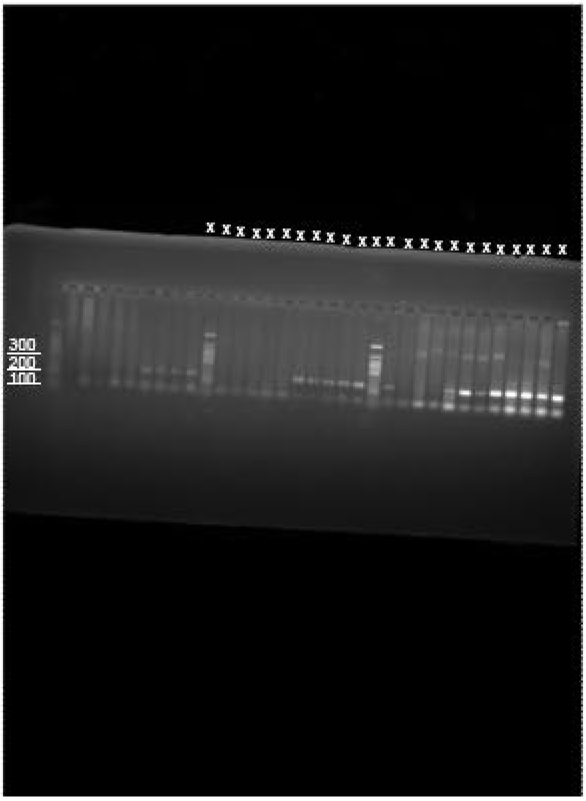

The image above represents P*salidodon fasciatus* BfMb-sbno2, corresponding to Figure 1b of the article. X=not related to the experiment.

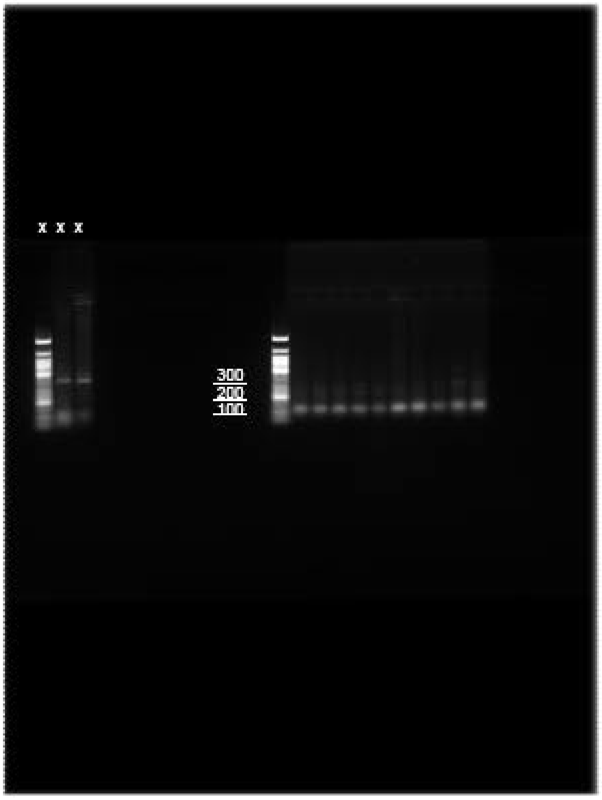

The image above represents *Psalidodon bockmanni* BbM-sbno2, corresponding to Figure 1b of the article. X is not related to the experiment.

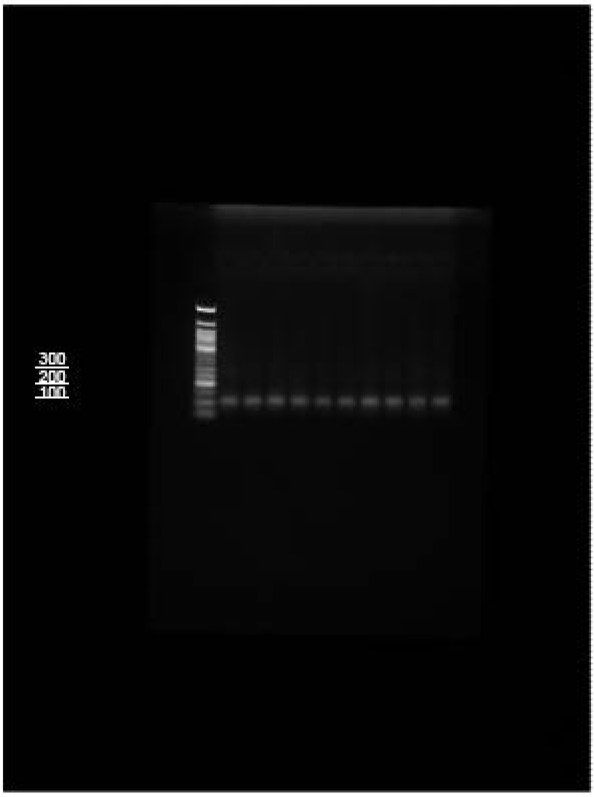

The image above represents *Psalidodon fasciatus* BfMa-simc1, corresponding to Figure 1b of the article. X=not related to the experiment.

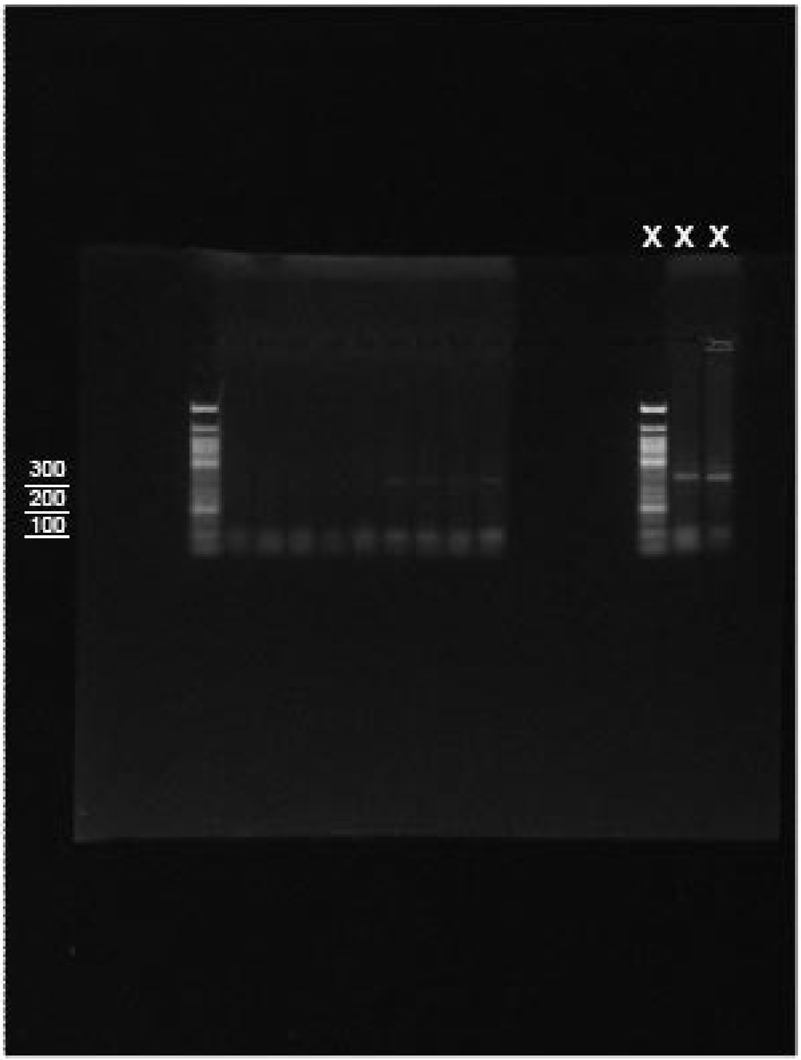

The image above represents *Psalidodon fasciatus* BfMb-simc1, corresponding to Figure 1b of the article. X=not related to the experiment.

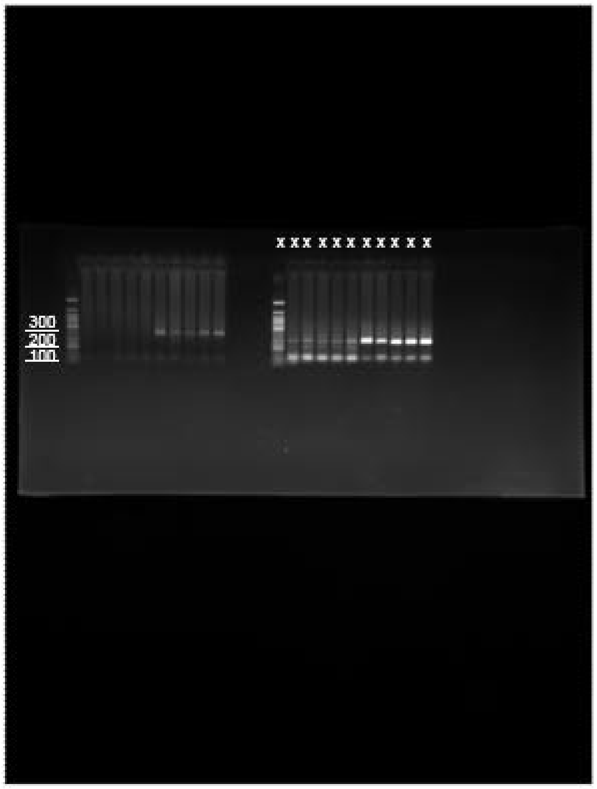

The image above represents *Psalidodon bockmanni* BbM-simc1, corresponding to Figure 1b of the article. X=not related to the experiment.

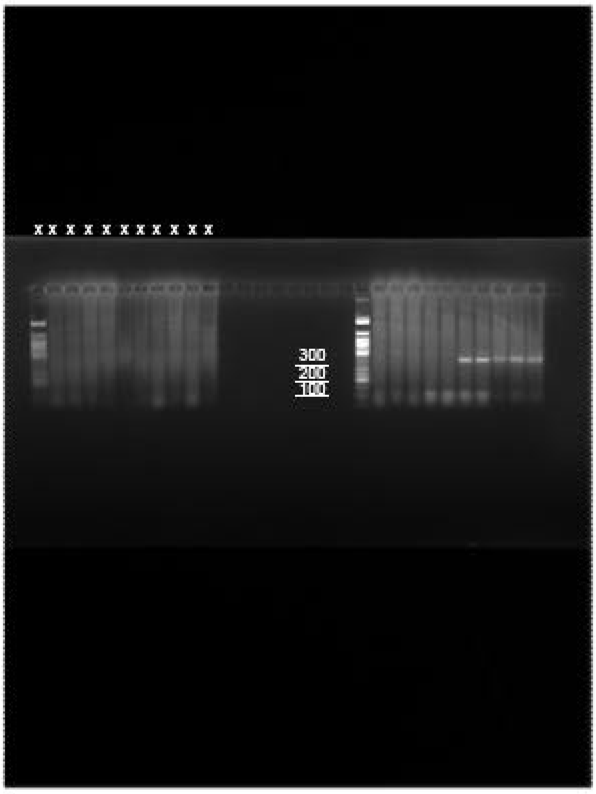

The image above represents *Psalidodon paranae* BpM-simc1, corresponding to Figure 1b of the article. X is not related to the experiment.

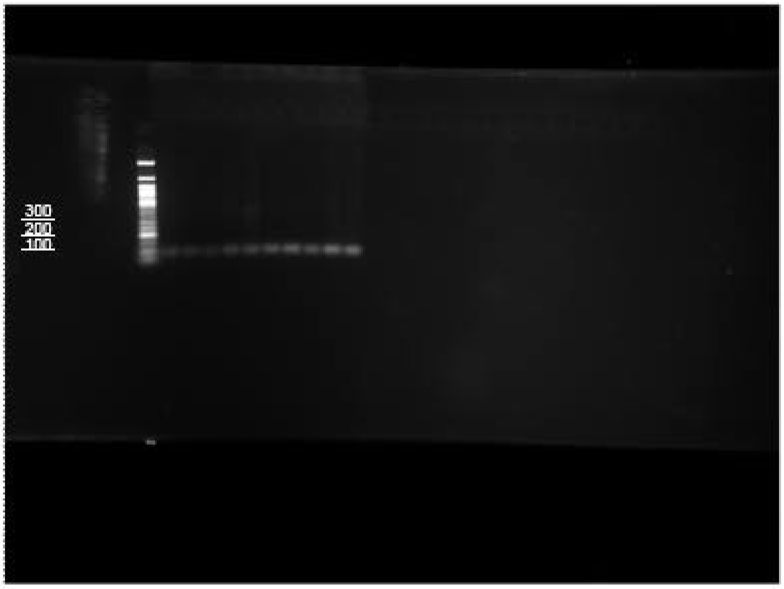

The image above represents *Psalidodon paranae* BpM-sbno2,exon-exon junction 9-10, corresponding to Figure 1b of the article.
X=not related to the experiment.
