# Supplementary material for: B chromosome retrotransposed sequences persist through speciation, contributing to genomic and regulatory innovations in the fish genus Psalidodon (Characiformes, Acestrorhamphidae)
Source: PLoS One. 2026 Jan 2;21(1):e0340085. doi: 10.1371/journal.pone.0340085 (PMC12758807; doi:10.1371/journal.pone.0340085)
Supplement: S1 File — (PDF) [file pone.0340085.s009.pdf]

Run statistics:

Number of input reads: 25706

Number of analyzed reads: 25706

Cluster merging: No

Consensus files - fasta format:

Supplementary Material S1. TAREAN clustering results for the sbno2 reads of Psalidodon paranae.

Documentation

For the explanation of TAREAN output see the help section

Putative satellites (high confidence)

not found

Putative satellites (low confidence)

not found

Putative LTR elements

not found

rDNA

not found

Other

	Cluster	Proportion[%]	Proportion adjusted[%]	Number of reads	Satellite probability	Consensus length	Consensus	Graph layout	TAREAN k-mer analysis	Connected component index C	Pair completeness index P	TAREAN k-mer coverage	|V|	|E|	PBS score	Similarity hits [above 0.1%]
1	1	11.000	11.000	2734	4.69e-21			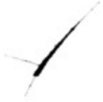	N/A	0.009880	0.07850		2734	1066844		
2	2	9.100	9.100	2331	1.37e-20			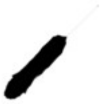	N/A	0.000429	0.06240		2331	1185271		
3	3	8.300	8.300	2137	1.27e-20			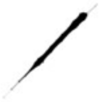	N/A	0.000468	0.04090		2137	701861		
4	4	6.900	6.900	1764	1.32e-20			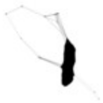	N/A	0.000567	0.04690		1764	766622		
5	5	6.800	6.800	1743	1.32e-20			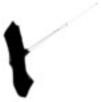	N/A	0.000574	0.04620		1743	705087		
6	6	6.700	6.700	1730	1.32e-20			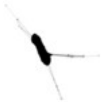	N/A	0.000578	0.04780		1730	716309		
7	7	6.300	6.300	1617	1.27e-20			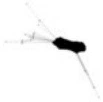	N/A	0.003090	0.03720		1617	722087		

8	8	6.200	6.200	1595	1.27e-20	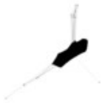	N/A	0.002510	0.04180	1595	670957
9	9	4.700	4.700	1205	1.78e-19	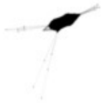	N/A	0.015800	0.03340	1205	488655
10	10	0.810	0.810	209	1.45e-19	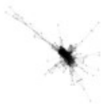	N/A	0.019100	0.00481	209	3921
11	11	0.300	0.300	78	5.03e-08	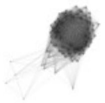	N/A	1.000000	0.01300	78	2412
12	12	0.250	0.250	64	1.45e-19	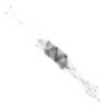	N/A	0.015600	0.00000	64	827
13	13	0.220	0.220	56	9.43e-09	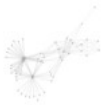	N/A	0.429000	0.00000	56	220
14	14	0.210	0.210	54	1.45e-19		N/A	0.018500	0.00000	54	208
15	15	0.180	0.180	47	1.67e-19	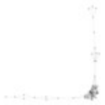	N/A	0.021300	0.02170	47	277
16	16	0.150	0.150	38	2.54e-19		N/A	0.026300	0.00000	38	164
17	17	0.130	0.130	33	2.54e-19	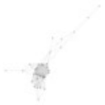	N/A	0.030300	0.00000	33	209
18	18	0.120	0.120	31	2.54e-19	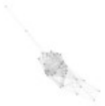	N/A	0.032300	0.00000	31	274
19	19	0.120	0.120	31	2.54e-19	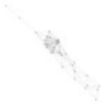	N/A	0.032300	0.00000	31	173
20	20	0.120	0.120	30	2.54e-19	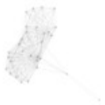	N/A	0.033300	0.00000	30	253

21	21	0.110	0.110	29	2.54e-19		N/A	0.034500	0.00000	29	120
22	22	0.093	0.093	24	4.85e-19		N/A	0.041700	0.00000	24	111
23	23	0.086	0.086	22	1.07e-18	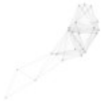	N/A	0.045500	0.00000	22	139
24	24	0.082	0.082	21	1.07e-18		N/A	0.047600	0.00000	21	63
25	25	0.078	0.078	20	1.07e-18		N/A	0.050000	0.00000	20	72
26	26	0.078	0.078	20	1.53e-18		N/A	0.050000	0.05260	20	70
27	27	0.078	0.078	20	1.53e-18		N/A	0.050000	0.05260	20	59
28	28	0.078	0.078	20	1.07e-18		N/A	0.050000	0.00000	20	73
