# Supplementary material for: B chromosome retrotransposed sequences persist through speciation, contributing to genomic and regulatory innovations in the fish genus Psalidodon (Characiformes, Acestrorhamphidae)
Source: PLoS One. 2026 Jan 2;21(1):e0340085. doi: 10.1371/journal.pone.0340085 (PMC12758807; doi:10.1371/journal.pone.0340085)
Supplement: S2 File — (PDF) [file pone.0340085.s010.pdf]

Supplementary Material S2. TAREAN clustering results for the simc1 reads of Psalidodon paranae.

Run statistics:

Number of input reads: 25104

Number of analyzed reads: 25104

Cluster merging: No

Consensus files - fasta format:

Documentation

For the explanation of TAREAN output see the help section

Putative satellites (high confidence)

not found

Putative satellites (low confidence)

not found

Putative LTR elements

not found

rDNA

not found

Other

	Cluster	Proportion[%]	Proportion adjusted[%]	Number of reads	Satellite probability	Consensus length	Consensus	Graph layout	TAREAN k-mer analysis	Connected component index C	Pair completeness index P	TAREAN k-mer coverage	|V|	|E|	PBS score	Similarity hits [above 0.1%]
1	1	9.300	9.300	2327	1.37e-20			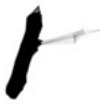	N/A	0.001720	0.0572		2327	1011265		
2	2	8.900	8.900	2231	1.27e-20			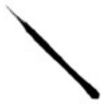	N/A	0.000448	0.0406		2231	643604		
3	3	8.600	8.600	2150	1.37e-20			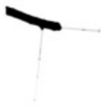	N/A	0.000465	0.0591		2150	797420		
4	4	8.000	8.000	2019	1.32e-20			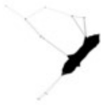	N/A	0.000495	0.0488		2019	888924		
5	5	6.400	6.400	1604	1.27e-20			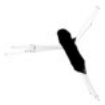	N/A	0.000623	0.0409		1604	656621		
6	6	6.300	6.300	1588	1.27e-20			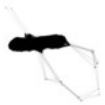	N/A	0.000630	0.0420		1588	564337		
7	7	6.100	6.100	1530	1.27e-20			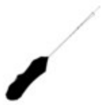	N/A	0.000654	0.0394		1530	594953		

8	8	5.600	5.600	1399	1.32e-20	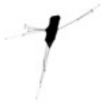	N/A	0.000715	0.0464	1399	535504
9	9	5.300	5.300	1322	1.27e-20	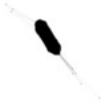	N/A	0.000756	0.0401	1322	538262
10	10	2.200	2.200	551	1.11e-20	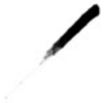	N/A	0.001810	0.0129	551	58591
11	11	0.530	0.530	133	2.35e-21	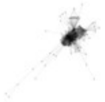	N/A	0.007520	0.0000	133	2130
12	12	0.370	0.370	92	2.23e-07	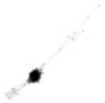	N/A	0.837000	0.0222	92	2200
13	13	0.230	0.230	57	1.90e-19	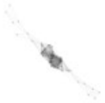	N/A	0.017500	0.0364	57	664
14	14	0.220	0.220	56	1.45e-19		N/A	0.017900	0.0000	56	185
15	15	0.200	0.200	50	1.45e-19		N/A	0.020000	0.0000	50	140
16	16	0.130	0.130	33	2.54e-19	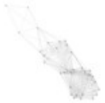	N/A	0.030300	0.0000	33	285
17	17	0.120	0.120	30	1.09e-07		N/A	0.500000	0.0000	30	99
18	18	0.100	0.100	25	4.85e-19		N/A	0.040000	0.0000	25	78
19	19	0.096	0.096	24	6.36e-19		N/A	0.041700	0.0435	24	177
20	20	0.096	0.096	24	4.85e-19		N/A	0.041700	0.0000	24	91

21	21	0.092	0.092	23	4.85e-19				N/A	0.043500	0.0000		23	164		
22	22	0.084	0.084	21	1.07e-18				N/A	0.047600	0.0000		21	107		
23	23	0.080	0.080	20	1.07e-18				N/A	0.050000	0.0000		20	57		
